# Enhancing Textile Water Repellency with Octadecyltrichlorosilane (OTS) and Hollow Silica Nanoparticles

**DOI:** 10.3390/polym15204065

**Published:** 2023-10-12

**Authors:** Mahshab Sheraz, Byul Choi, Juran Kim

**Affiliations:** 1 Advanced Textile R&D Department, Korea Institute of Industrial Technology (KITECH), Ansan 15588, Republic of Korea; mahshab@kitech.re.kr (M.S.); bychoi@kitech.re.kr (B.C.); 2 HYU-KITECH Joint Department, Hanyang University, 222, Wangsimni-ro, Seongdong-gu, Seoul 04763, Republic of Korea

**Keywords:** eco-friendly superhydrophobic coating, PET fiber, porous hollow silica nanoparticles (HS), water repellency, textile applications

## Abstract

Superhydrophobic coatings have attracted substantial attention owing to their potential application in various industries. Conventional textiles used in daily life are prone to staining with water and household liquids, necessitating the development of water-repellent and stain-resistant coatings. In this study, we fabricated a highly water-repellent superhydrophobic PET fabric by using an eco-friendly water-based coating process. Fluorine-free octadecyltrichlorosilane (OTS) solutions with various wt.% of hollow silica (HS) nanoparticles were used to produce a superhydrophobic surface via a facile dip coating method. Our findings revealed that the incorporation of HS nanoparticles substantially increased the water contact angle, with higher concentrations resulting in enhanced water repellency and increased surface roughness. The treated fabrics had a remarkable water contact angle of 152.4° ± 0.8°, demonstrating their superhydrophobic fiber surface. In addition, the durability of these superhydrophobic properties was investigated via a laundry procedure, which showed that the fabrics maintained their water repellency even after 20 laundering cycles. EDX and XRD analyses confirmed that the morphological evaluations did not reveal any substantial structural alterations. Significantly, the fibers maintained their strength and durability throughout the testing, enduring only minor hollow SiO_2_ nanoparticle loss. This eco-friendly and cost-effective method holds great potential for application in apparel and other industries, offering an effective solution to resist water stains and improve performance in various contexts.

## 1. Introduction

Superhydrophobicity is a frequently observed phenomenon in natural structures such as rose petals, rice leaves, lotus leaves, and butterfly wings. This has been a focal point of substantial research and innovation efforts. Applications of superhydrophobic surfaces have been explored across diverse fields, offering a multitude of advantages. These benefits include self-cleaning capabilities, resistance to corrosion, the mitigation of fouling and fogging, and the capacity to effectively separate oil and water, decrease fluid drag, and improve compatibility with blood [1,2,3]. There has been a significant surge of interest in the development of technologies for producing superhydrophobic surfaces due to their potential benefits in various applications [4,5]. The scientific literature indicates that a material’s ability to exhibit superhydrophobic properties is influenced by both its surface chemistry and its surface structure [6]. Two distinct theoretical models, namely the Wenzel and Cassie–Baxter models, have been applied to facilitate the fabrication of superhydrophobic surfaces [7]. This achievement has been attained through techniques such as surface roughening, reducing surface-free energy, or a combination of both approaches.

Superhydrophobic surfaces hold significant potential for application across a wide spectrum of industries [8,9]. Recently, the growing market demand for high-performance textiles has presented challenges and opportunities for finishers globally [10]. The expansion of the casual and sportswear markets has led to an increasing need for superhydrophobic fabrics. Such fabrics exhibit a water contact angle of 150° or higher with a practically non-wettable superhydrophobic surface [9]. Consequently, the market demand for superhydrophobic fabrics has increased daily. Various methods are available for achieving superhydrophobicity in textiles, such as the use of low surface energy compounds, formaldehyde, and fluorinated compounds. Table S1 offers a consolidated overview of the diverse methodologies used to achieve superhydrophobic surfaces, enhancing their ability to repel water effectively. The majority of prior research has focused on the application of fluorochemicals owing to their remarkable water-repellent properties. Nonetheless, the utilization of fluoroalkyl compounds has notable drawbacks, including their elevated cost and the potential hazards they pose to human health and the environment [11,12,13,14]. Fluorinated compounds are frequently employed as hydrophobic agents because of their advantageous attributes, including their reduced surface energy compared to various other compounds (-CH_2_ > -CH_3_ > -CF_2_ > -CF_2_H > -CF_3_), inherent oleophobic properties, resistance to chemical interactions, and stability across a wide temperature range, encompassing both high and low temperatures [15,16,17]. However, it is important to note that long-chain perfluorinated alkyl substances, which belong to the category of fluorinated compounds, can lead to bioaccumulation and toxicity, posing risks to both human health and the environment [18,19]. Unfortunately, these materials are currently subject to the regulation of hazardous substances (RoHS). By 2025, the European Union (EU), the United States of America (USA), and Japan will enforce prohibitions on these materials. Hence, there is an immediate need to identify alternative non-fluorinated modifying agents to develop environmentally friendly hydrophobic textile materials. Consequently, octadecyltrichlorosilane (OTS) has garnered significant attention from researchers as an excellent reagent for coating production. OTS is a widely employed organosilane derivative that offers the advantage of requiring only water and a solvent such as hexane for the coating fabrication process. Octadecyltrichlorosilane (OTS) is extensively manufactured and is recognized for its ability to alter the surface properties of various solid substrates through the formation of densely packed and highly oriented self-assembled monolayers (SAMs). Furthermore, the absence of fluorine contributes to a reduction in environmental and health-related risks associated with its usage [20,21,22].

Surface energy and surface roughness are crucial factors for achieving a water contact angle (CA) exceeding 150° [23,24,25,26,27]. When a material possesses the lowest possible surface energy, it can only attain a water contact angle of approximately 120°, indicating either a degree of hydrophobicity or excellent hydrophobic properties [28]. To attain higher levels of hydrophobicity (superhydrophobicity), it is necessary to increase surface roughness [29]. Extensive research efforts have been dedicated to creating superhydrophobic surfaces, with a particular focus on sol-gel methods. Within this context, various inorganic nanoscale particles such as TiO_2_ [23], ZnO [25,30], graphene [31], carbon nanotubes (CNTs) [32], and indium tin oxide (ITO) [33] have been used to enhance the surface roughness of materials. More recently, SiO_2_ sol and SiO_2_ nanoparticles have garnered significant attention from researchers due to their capacity to generate superhydrophobic surfaces [3,9]. This capability can be attributed to their unique capacity to yield nanostructures, facilitate surface modification, maintain optical transparency, and exhibit remarkable thermal stability. Furthermore, these nanoparticles agglomerate on polymer surfaces, giving rise to an ideal hierarchical morphology characterized by intricate micro- and nanoscale rough structures [34]. There are several published research articles in which silica sol was used to increase surface roughness [9,35,36], but very few articles in which silica nanoparticles were used. It has been reported that silica nanoparticles can increase the surface roughness and the adhesion stability of silica nanoparticle-based superhydrophobic coatings, addressing one of the long-standing issues for nanoparticle-based liquid-repellent coatings. This enhanced adhesion assures the durability and longevity of the superhydrophobic properties [37].

In this study, we have undertaken a pioneering approach by incorporating the latest advancements in modified hollow silica (HS) nanoparticles. Our primary objective was to synthesize and apply these nanoparticles to augment surface roughness. In response to the growing environmental concerns regarding fluorine-based compounds, we developed environmentally friendly water-repellent formulations by integrating non-fluorine-based compounds with OTS and HS nanoparticles. Superhydrophobic fabrics were fabricated using a cost-effective and straightforward dip-coating method on a polyethylene terephthalate (PET) fabric substrate. This novel methodology was designed to establish a synergistic effect on fabric surfaces, leading to substantial enhancement in their water-repellent properties. Subsequently, we rigorously tested the prepared fabric and subjected it to laundering in a washing machine to evaluate its durability. This critical step ensures that the fabric is well suited for commercial applications and everyday use as water-repellent textiles.

## 2. Experimental Section

### 2.1. Materials and Methods

We procured high-quality chemicals from Sigma–Aldrich (Seoul, Republic of Korea), which included octadecyltrichlorosilane (OTS) (≥90%) and hexane (≥97.0% as confirmed by GC). We received a shipment from Bosungtex in (Seoul, Republic of Korea), consisting of 100% polyester textiles in a white color, with the model number BE-BM6910NZ. To obtain deionized water with a resistivity exceeding 18.2 MΩ cm, we employed a Barnstead EasyPure UV/UF compact water system (Model No. D8611, Dubuque, IA, USA). We acquired extra pure Sodium silicate (Na_2_SiO_3_·9H_2_O) (>99.9%), calcium carbonate (CaCO_3_) (>99.0%), hydrochloric acid (HCl) (>99.0%), rose Bengal dyes (>99.0%), and purified ethyl alcohol (>99.5%) (C_2_H_6_O) from Sigma–Aldrich and utilized them without any further need for purification.

### 2.2. Preparation of Hollow Hydrophobic SiO_2_ Nanoparticles

Hollow silica (HS) nanoparticles were synthesized using a step-by-step procedure. Initially, sodium silicate was gradually added to a suspension of nanosized calcium carbonate while the suspension was maintained at 80 °C using a thermostatic water bath. The pH of the mixture was adjusted to within the range of 9–10 by adding 10 wt.% hydrochloric acid (HCl) solution. After the mixture was stirred for 2 h, a composite with a SiO_2_:CaCO_3_ molar ratio of 1:10 was formed. The composite was then filtered and washed with ethanol and distilled water. The resulting solid was then dried at 100 °C and calcined at 700 °C for 5 h, leading to the formation of a core-shell compound consisting of CaCO_3_ and SiO_2_. The CaCO_3_ template was completely removed by immersing the compound in a 10 wt.% dilute solution of HCl overnight. The resulting gel was filtered, washed, and subjected to another round of calcination at 100 °C for 24 h. This final step successfully yielded the HS nanoparticles.

### 2.3. Preparation of Superhydrophobic Solution for Textile Coating 

The coating solution was prepared following the optimal conditions established in a previous study [19]. 200 μL of water was added to a 50 mL centrifuge tube containing pure OTS (10.0 mL), and the mixture was immediately shaken at 3200 rpm for 10 s using a vortex mixer and a tube cap. Subsequently, the tube was sonicated in an ultrasonic cleaner for 10 s without a cap, followed by an additional 10 s mixing steps on the vortex mixer while capped. Next, 5000 μL of the resulting solution was transferred to a 200 mL glass vial, which was closed with a cap but not sealed tightly. After allowing the solution to settle for 2 h, 100 mL of hexane was added to the vial. The mixture was stirred using a magnetic stirrer for 2 h before use. This process was repeated multiple times to prepare coating solutions in separate glass vials.

**Figure 1:**
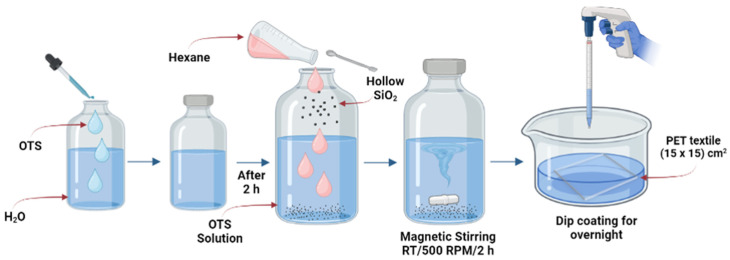
Schematic of the manufacturing process for superhydrophobic surfaces incorporating hollow silica (HS) powder modified with octadecyltrichlorosilane (OTS).

HS powder was added into each glass vial at different weight ratios (0.5, 1.0, 1.5, and 2.0 wt.%) according to the pure OTS content. The mixtures were stirred for 2 h before application using a magnetic stirrer. To obtain superhydrophobic surfaces, PET fabric substrates (15 × 15 cm^2^) and glass slides (5 × 5 cm^2^) were prepared. The substrates were then treated by overnight immersion in the prepared coating solutions using the dip-coating method. Figure 1 visually illustrates the entire experimental process. After immersion, the samples were removed from the solutions, washed three times with hexane, and left to dry in ambient air for 5 h. Notably, the glass slides underwent an additional step of cleaning with a UV-Ozone cleaner for 30 min.

### 2.4. Characterization and Instrumentation 

The contact angles of the fabric samples—the untreated PET fabric (UT), the PET fabrics coated with OTS (PO), the glass coated with OTS (GO), the PET fabrics coated with OTS and 0.5 wt.% HS [PO-HS(0.5)], the PET fabrics coated with OTS and 1.0 wt.% HS [PO-HS(1.0)], the PET fabrics coated with OTS and 1.5 wt.% HS [PO-HS(1.5)], and the PET fabrics coated with OTS and 2.0 wt.% HS [PO-HS(2.0)]—were determined using a drop shape analyzer (DSA25, Kruss, Germany) and the sessile drop technique at room temperature. A 6 μL droplet of deionized water (surface tension (γLV) = 72.8 mN/m) was deposited on each fabric sample using a syringe, and the measurement was repeated six times at various locations. Fourier transform infrared spectroscopy (FTIR) was employed to analyze the samples using either a Nexus670 (Gaithersburg, MD, USA) or a Nicolet IS50 (Thermo Fisher Scientific, Waltham, MA, USA). The FTIR spectra were recorded in the range of 500–4000 cm^−1^ at a controlled ambient temperature of 25 °C. The specimens were scanned from 4000 to 400 cm^−1^ with a resolution of 2 cm^−1^, averaging 128 scans. The morphology and microstructure of the samples were analyzed through a combination of techniques, including field emission scanning electron microscopy (FESEM) using a Hitachi instrument from Tokyo, Japan, Energy Dispersive X-ray (EDX) analysis, and transmission electron microscopy (TEM) at 200 kV. The particle size was assessed by employing the Image J software (version java 1.8.0), a tool developed by the National Institutes of Health (NIH), through the analysis of Field Emission Scanning Electron Microscopy (FESEM) and Transmission Electron Microscopy (TEM) images. We conducted X-ray diffraction (XRD) analysis using the Bruker D8 Advance instrument located in Billerica, MA, USA. The objective of this technique was to examine the morphology of both untreated and treated fibers and assess any morphological alterations in the fibers before and after undergoing washing cycles. Thermogravimetric analysis (TGA) was performed using a Thermogravimetric Analyzer (TGA Q500, TA Instruments, New Castle, DE, USA) to assess thermal stability. The TGA measurements were conducted under a nitrogen atmosphere from 25 to 800 °C at a heating rate of 20 °C/min. To evaluate the resistance to surface wetting, a mechanical performance test was performed using a washing machine, and water repellency was assessed before and after washing. The washing machine test was performed 20 consecutive times at 40 °C for 30 min each time, followed by the hot air drying of the samples.

### 2.5. Water Contact Angle (WCA) and Washing Resistance (WR) Assays

The hydrophobic properties of the prepared fabrics were evaluated using water contact angle (WCA) measurements, and their durability was assessed by washing. The WCA was measured using an optical video contact angle instrument (DSA 25, Kruss, Hamburg, Germany) under standard room-temperature conditions. The presented CA values were obtained by calculating the mean of measurements taken at five distinct locations on each fabric sample. The testing and evaluation of the laundering durability of the chemically modified fabrics were conducted according to the guidelines outlined in AATCC Test Method 61-2003, as recommended by the American Association of Textile Chemists and Colorists. The experiment was conducted using a conventional accelerated laundering apparatus outfitted with 200 mL stainless-steel lever-lock canisters. The water temperature was maintained at 24 °C. The treated PET fabric (20 × 20 cm^2^) was subjected to a laundering process, in which it was immersed in a solution consisting of 5 wt.% Tide liquid laundry detergent dissolved in water. The solution was stirred at 150 rpm and maintained at 24 °C for 60 min. After washing, the fabrics were rinsed with water and air dried for 12 h at 24 °C.

## 3. Results and Discussion

### 3.1. Superhydrophobic Surface: Reaction Mechanism and Surface Morphology

**Scheme 1:**
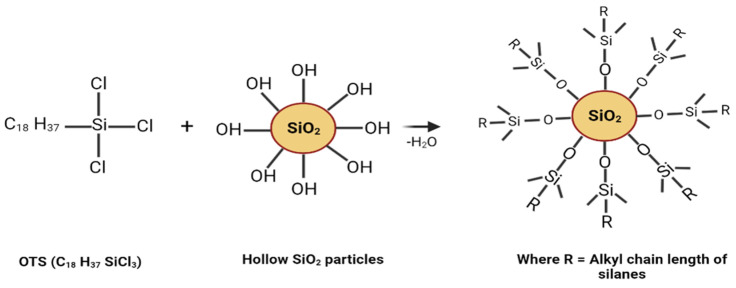
Depicts the reaction between silica and trichloro(alkyl)silane, resulting in the formation of alkylsiloxane-silica.

**Figure 2:**
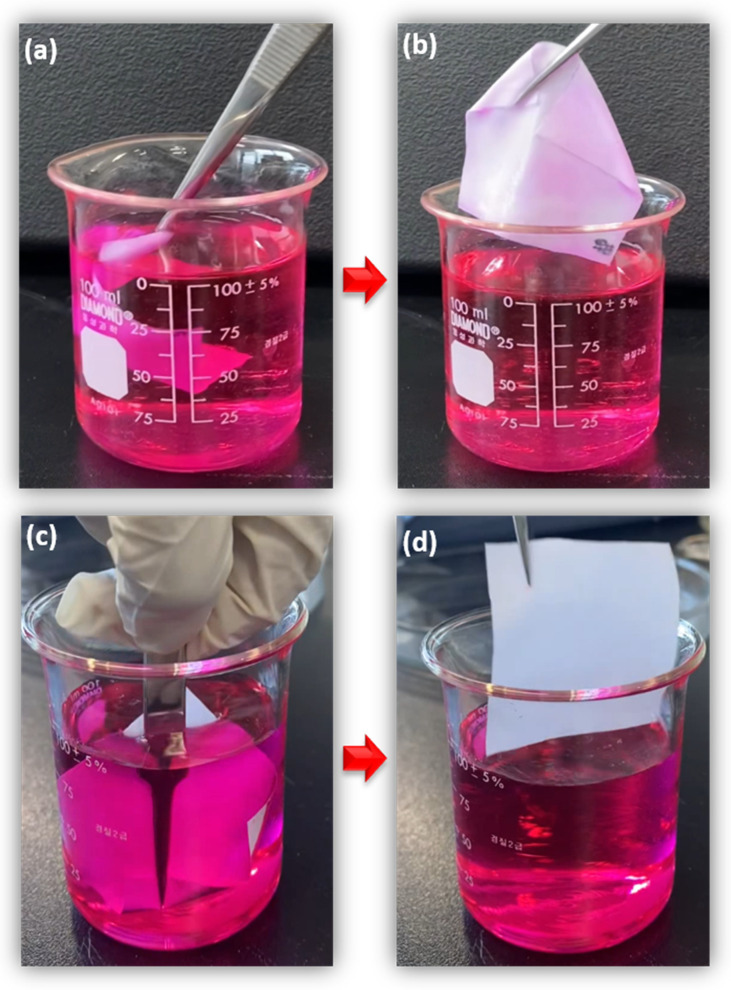
Comparison of untreated PET fabric and PET fabric treated with a special coating showing differences when immersed in a pink solution. (**a**) Untreated fabric immersed in the solution (**b**) appearance of untreated fabric after immersion (**c**) Coated fabric immersed in the solution (**d**) appearance of the coated fabric after immersion.

The experimental procedure presented in Figure 1 involves the modification of hollow silica nanoparticles using OTS to impart superhydrophobicity. The superhydrophobic silica nanoparticles are applied to the OTS-coated PET fabric using a simple dip-coating method where the porous PET fabric is filled with interconnected silica nanoparticles, resulting in a durable superhydrophobic textile. In Scheme 1, the silica undergoes a modification process using organosilane (OTS) to achieve superhydrophobic properties. During the coating procedure, the PET fabric becomes densely packed with silica nanoparticles networked through alkylsiloxane, resulting in the fabrication of a robust superhydrophobic PET membrane. This transformation is facilitated by the chemical bonding formed through the reaction between trichloro(alkyl)silane and the surface hydroxyl groups (OH) (silanols) present on the silica nanoparticles. The chemical structure of OTS, composed of an octadecyl (C18) hydrocarbon chain linked to a trichlorosilane (SiCl_3_) group, plays a pivotal role in this process. The hydrophobic characteristics stem from the hydrocarbon chain, while the trichlorosilane group enables the attachment of the OTS molecule to various surfaces, including silica nanoparticles. The pristine and prepared samples were labeled as follows: HS powder, UT, PO, and PO-HS(0.5), PO-HS(1.0), PO-HS(1.5), and PO-HS(2.0). The characteristics of the samples are listed in Table 1. The UT PET fabric exhibited a pronounced hydrophilic nature as it readily absorbed water. The transformation of the fabric before and after immersion in the solution is depicted in Figure 2. Upon immersion, the fabric became thoroughly wet, and its color changed to pink, indicating the high affinity of the UT fabric for water. A detailed visual representation of the immersion process is provided in Supplementary Movie S1. In contrast, the PET fabric treated with OTS and augmented with HS nanoparticles PO-HS(2.0) exhibited significantly increased superhydrophobicity. This pronounced improvement is presented in Figure 2, which shows the state of the fabric before and after immersion in the solution. After immersion, the fabric exhibited remarkable superhydrophobic behavior. Supplementary Movie S2 provides a comprehensive visual representation of this process and elucidates the observed transformations. Because of the hydrophobic nature of the OTS coating, the prepared fabric exhibited excellent water-repellent characteristics and a non-sticky surface. This water-repellency prevents the fabric from absorbing water or being wetted by aqueous solutions, therefore keeping it dry when immersed in water with 0.01 mmol rose Bengal dyes used for easy identification [19]. Such results demonstrate the superhydrophobicity of the OTS-coated PET fabric and highlight its potential for various applications requiring water resistance and repellency. The thickness of the superhydrophobic PET fiber coated with PO-HS(2.0) was measured using a micro-hite instrument (TESA µ-Hite 07.30049) manufactured by Swiss TESA in Renens, Switzerland. The measurement revealed that the fiber thickness was 0.10 mm.

**Figure 3:**
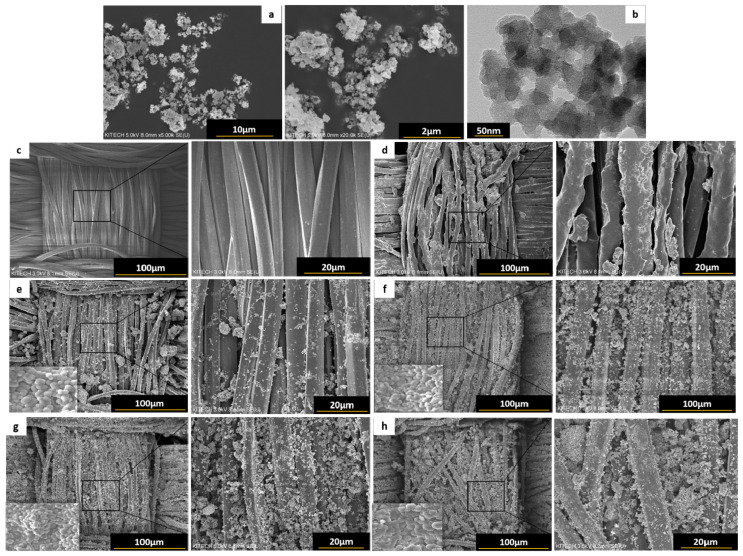
(**a**) Field emission scanning electron microscopy (FESEM) and (**b**) transmission electron microscopy (TEM) images of HS nanoparticles revealing the surface morphologies of the samples. FESEM images of (**c**) untreated (UT) polyethylene terephthalate (PET) fabric, (**d**) PET fabric coated with octadecyltrichlorosilane (OTS) solution (PO), (**e**) PO-HS(0.5), (**f**) PO-HS(1.0), (**g**) PO-HS(1.5), and (**h**) PO-HS(2.0).

The surface microstructures of the specimens and the morphologies of the HS nanoparticles were analyzed using FESEM and TEM with different magnifications Figure 3a,b. The nanoparticles tended to aggregate into asymmetrical lumps, which can be attributed to their large specific surface areas [38]. SiO_2_ nanoparticles are hollow and have a significant internal surface area that renders them suitable for substrate attachment [39]. The mean diameter of the SiO_2_ nanoparticles was determined to be 36.68 nm. The nanoparticles can be seen with hollow structures with smooth surfaces. The distribution of particle sizes is visually represented in Figure S1. The analysis of the UT PET fabric specimen revealed a multitude of fiber strands that were interlaced in both the warp and weft directions, exhibiting a high degree of alignment and compactness Figure 3c. The PO exhibited a uniform coating of each fiber strand Figure 3d owing to the interaction of the agglomerated OTS solution with a small amount of water. Figure 3e–h shows the surface morphologies of the PET fibers coated with the OTS solution and treated with SiO_2_. The FESEM image shows the existence of abundant silicon dioxide (SiO_2_) nanoparticles on the surface of the fiber, resulting in a coarse and uneven visual aspect. The nanoparticle distribution is uniform, forming a compact layer on the fiber surface. The surfaces of the PO-HS(0.5)–PO-HS(2.0) samples exhibited the adherence of fine spherical nanoparticles of HS powder to the fiber strands, as shown in Figure 3e–h. Furthermore, with an increase in the weight proportion of the incorporated HS powder, the distribution of the nanoparticles increased. It has been noted that the fiber strands are enveloped with a larger amount of coating solution. The complete coverage of the fiber surface by the modified silica nanoparticles was apparent, which produced a rough surface with low free energy, rendering it water-repellent.

### 3.2. Chemical Properties

**Figure 4:**
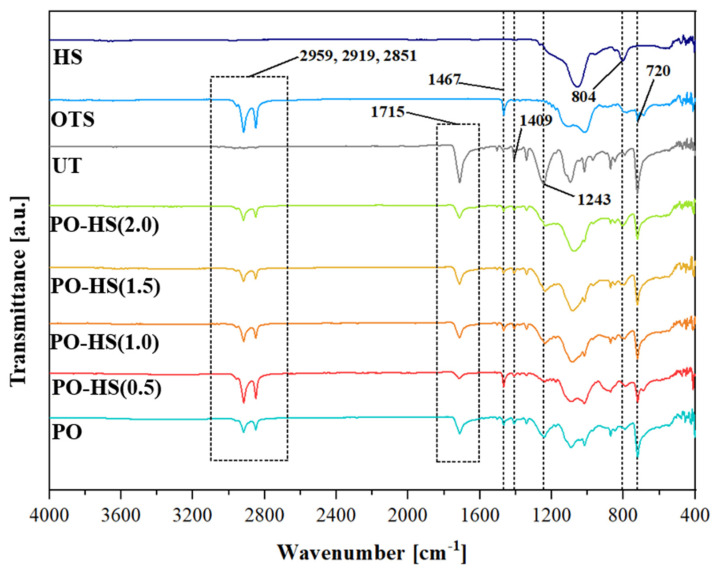
FTIR spectra of hollow silica nanoparticles (HS), octadecyltrichlorosilane (OTS), and PET fabric coated with OTS solution (PO). Surface modification through the incorporation of different weight percentages of (HS) nanoparticles, resulting in the formation of PO-HS(0.5), PO-HS(1.0), PO-HS(1.5), and PO-HS(2.0).

The chemical properties of the samples were analyzed using Fourier-transform infrared (FT-IR) spectroscopy (Nexus670, Gaithersburg, MD, USA). Figure 4 in the range of 400–4000 cm^−1^. FT-IR allowed the identification and examination of specific molecular vibrations associated with the OTS-coated PET fabric, providing valuable insights into its chemical structure and composition. The absorption bands at 2851 and 2919 cm^−1^ are attributed to symmetric and asymmetric stretching modes, respectively, and the -CH_2_ groups (methylene groups) are present in the long-chain alkyl group of the OTS compound [40,41]. The peak at 2959 cm^−1^ can be attributed to the asymmetric stretching of the -CH_3_ groups present in OTS. Additionally, the absorption band at 1715 cm^−1^ indicates the vibrational motion of C=O bonds, specifically associated with carbonyl groups. The absorption peaks at 1243 and 1096 cm^−1^ suggest the presence of C-C-O and O-C-C linkages, respectively. Furthermore, the peak at 722 cm^−1^ signifies the presence of C-H bonds, while the peak at 1409 cm^−1^ can be attributed to the characteristic vibration of aromatic rings, which is indicative of the presence of polyester [42]. The peak at 804 cm^−1^ can be attributed to the asymmetric stretching vibration band of Si-O-Si, suggesting the presence of HS nanoparticles on the OTS-coated PET fabric. The summary of FTIR results is presented in Table S2.

### 3.3. Thermal Properties 

**Figure 5:**
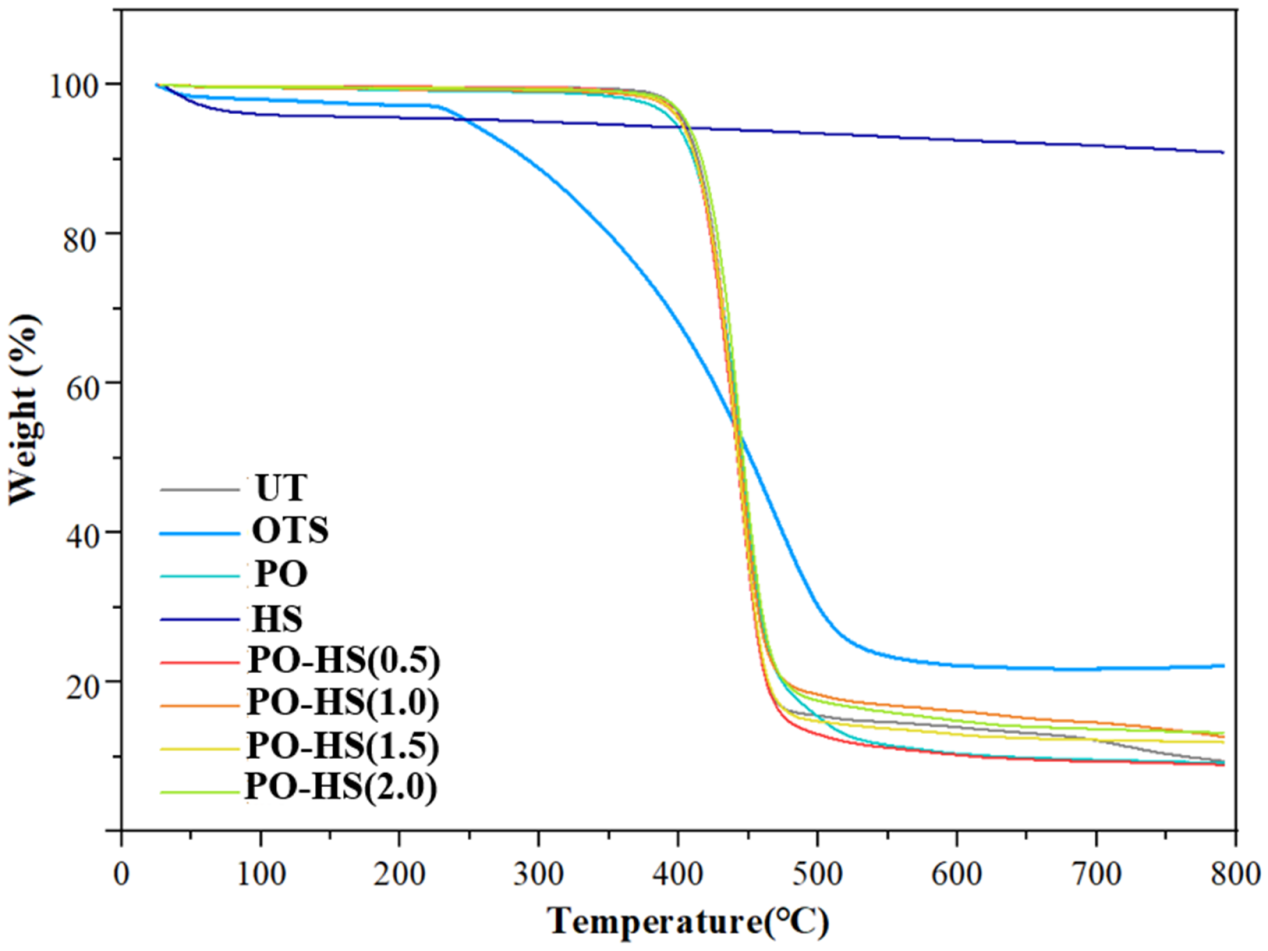
TGA results indicating the rates of thermal degradation under ambient air conditions of untreated (UT) PET fabric, octadecyltrichlorosilane (OTS), PET fabric coated with OTS solution (PO), and hollow silica nanoparticles (HS) incorporated at varying weight percentages. The introduction of HS nanoparticles results in the formation of superhydrophobic fabric surfaces, specifically labeled as PO-HS(0.5), PO-HS(1.0), PO-HS(1.5), and PO-HS(2.0), respectively.

The thermal stability of all samples was assessed using TGA, and the results are presented in Figure 5. The weight loss of the HS sample exhibited minimal variation when exposed to a temperature as high as 800 °C. This observation indicates that the HS nanoparticles have remarkable thermal stability, as they can maintain their weight and structure without substantial degradation or decomposition even under high-temperature conditions [43,44,45,46]. The UT PET sample exhibited a rapid decrease in weight at approximately 492 °C during TGA analysis. This weight loss indicates that the untreated PET fabric began to undergo significant decomposition or degradation at this temperature. However, the weight of the sample treated with OTS decreased by approximately 556 °C. This weight loss suggests that the OTS-coated PET fabric (PO) began to decompose or degrade at a slightly higher temperature than the untreated PET. Such findings indicate that the OTS treatment enhanced the thermal stability of the PET fabric, as evidenced by the delayed onset of weight loss compared with that of the untreated sample. The OTS coating likely offered protection against thermal degradation, leading to a higher temperature threshold for weight loss. The samples treated with the OTS solution and SiO_2_ nanoparticles, particularly PO-HS(1.0) and PO-HS(2.0), exhibited a weight loss at a temperature near 556 °C, as observed in the TGA curves. The observed temperature was marginally greater than the thermal stability demonstrated by the UT PET sample. The OTS coating was expected to have a protective effect, leading to the postponement of weight loss upon exposure to higher temperatures.

### 3.4. Wettability and Durability Test

#### 3.4.1. Wettability and Water Contact Angle (WCA)

To assess the wettability of the OTS-coated PET fabric modified with silica nanoparticles, the water with 0.01 mmol rose Bengal dyes was dropped onto the fabric surface. Supplementary Movie S3 presents the interaction of water droplets with the treated surface; the water droplets easily bounce off the treated surfaces upon contact. After several bounces, the droplet eventually leaves the surface without leaving any stains or residues. This behavior indicates the excellent water repellency and superhydrophobic nature of the OTS-coated PET fabric.

**Figure 6:**
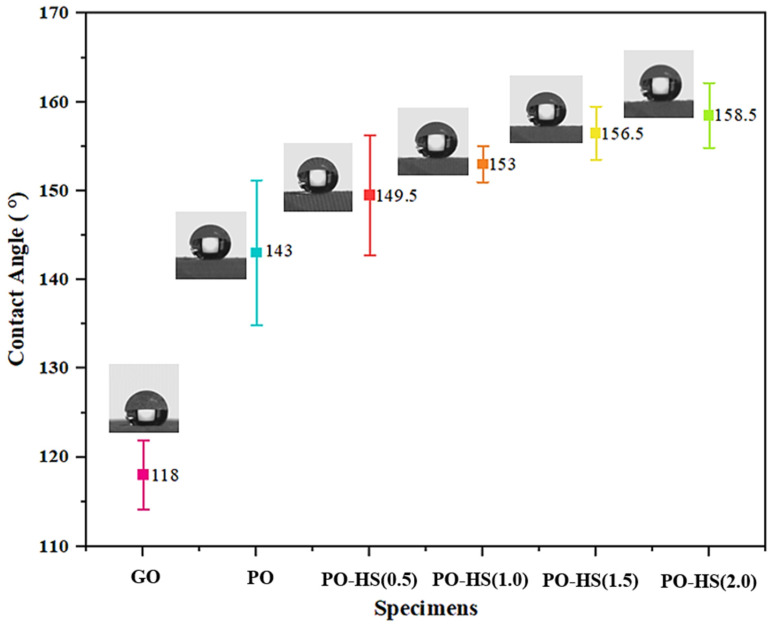
Static water contact angles (WCAs) of GO, PO, PO-HS(0.5), PO-HS(1.0), PO-HS(1.5), and PO-HS(2.0).

The WCA serves as a crucial parameter in assessing the hydrophobicity of a surface. It is determined by measuring the angle between the surface and the tangent lines at the modified circumference of a water droplet [47]. Previous research has demonstrated that an OTS solution resulted in the formation of a hierarchical microstructure on the surface and reduced surface tension through stoichiometric reactions with water. In the present study, the surface irregularity increased as the proportion of porous HS powder increased, which directly affected the WCA results [19]. To compare water repellency, the WCAs of different samples were measured, as shown in Figure 6. The average WCAs of the GO and PO samples coated with only the OTS solution (without the addition of HS) were 118° and 143°, respectively. In contrast, the WCAs of PO-HS(0.5), PO-HS(1.0), PO-HS(1.5), and PO-HS(2.0) treated with the OTS solution containing HS were 149.5°, 153°, 156.5°, and 158.5°, respectively. These results indicate that the PO-HS(0.5)–PO-HS(2.0) samples exhibited higher water repellency than GO and PO, exhibiting superhydrophobic properties. Notably, the WCAs increased proportionally with the amount of HS powder, with HS4 exhibiting the highest WCA. These findings are consistent with the TGA results, where HS4 showed the highest residue content (13.3%) at 790 °C among the samples treated with the OTS solution PO, HS(0.5)–HS(2.0). The increase in the WCA and residue (%) highlights the substantial contribution of HS to the enhancement of water repellency.

Furthermore, it should be noted that the WCA of GO on the glass substrate was significantly lower than that of the samples on the treated PET substrate PO and all samples coated with OTS and HS (0.5–2.0). This difference can be attributed to the absence of pores on the glass surface, which limits the coating solution to the surface and reduces surface roughness. These results highlight the role of HS in increasing water repellency and support the hypothesis that the addition of HS contributes to the observed superhydrophobic properties. The WCA and residue (%) values are summarized in Table 2, providing a comprehensive overview of the relationship between the HS content, water contact angle, and residue formation.

#### 3.4.2. Laundering Durability of the Treated Fabric 

**Figure 7:**
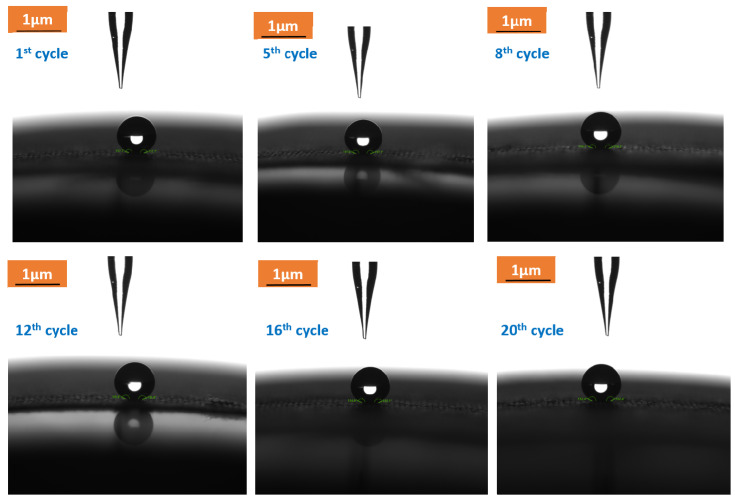
Shows the assessment of superhydrophilicity durability using water contact angle measurements after 20 laundering cycles for the optimal PO-HS (2.0) sample.

For practical and real-life applications, textile fabrics must exhibit durable water repellency, even after multiple laundering cycles. To assess the resistance of the prepared fabric samples to laundering cycles, their hydrophobicities were examined. As previously discussed, the PO-HS(2.0) sample exhibited the highest WCA, indicating superior water repellency. Consequently, further experiments were conducted using PO-HS(2.0) to evaluate its performance after 20 home laundering cycles. The changes in WCA with respect to the number of laundering cycles are shown in Figure 7. After 20 wash cycles, the WCA of the treated PET fabric decreases from 158.3° to 152.4°. Notably, this reduction was minimal, indicating a relatively small decline in water repellency. Moreover, it is worth mentioning that the treated PET fabric consistently maintained a WCA above the hydrophobic threshold of 90°. Table 3 provides additional insights, demonstrating that most of the hydrophobic properties decreased after the 12th laundering cycle. Subsequently, the decrease in the WCA became negligible, indicating the stable water repellency of the fabric until the 20th laundering cycle.

#### 3.4.3. Morphological Transformation before and after Laundering of PO-HS(2.0) Fiber

**Figure 8:**
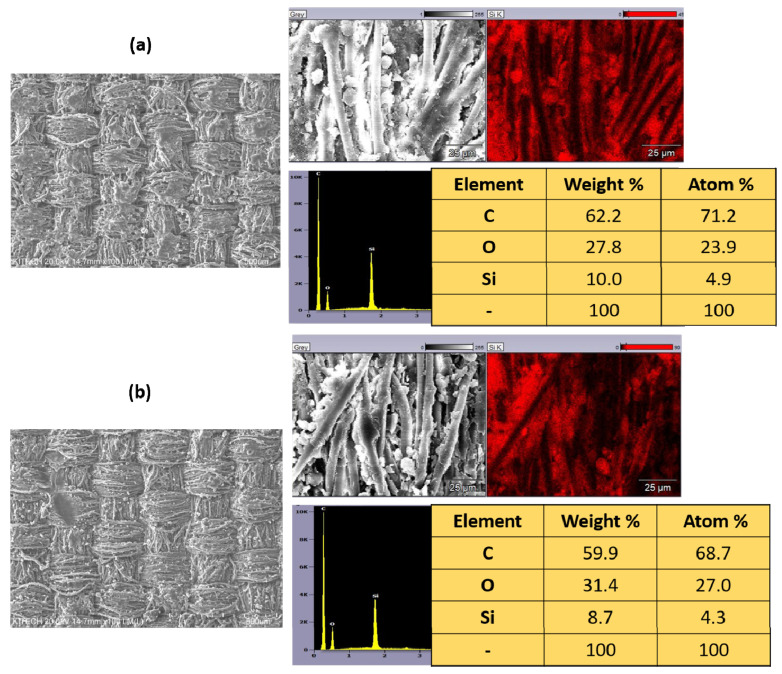
Illustrates critical facets of the study. (**a**) presents the FESEM image and corresponding EDX spectra, providing an overview of the elemental distribution within the PO-HS(2.0) fiber before any washing procedures. In contrast, (**b**) displays the FESEM image and EDX spectra of the same PO-HS(2.0) fiber after undergoing 20 successive laundering cycles.

This study involved a comparison of PO-HS (2.0) fabric samples before and after the washing process to examine any morphological changes using Field Emission Scanning Electron Microscopy (FESEM) (Hitachi, Tokyo, Japan) and Energy Dispersive X-ray (EDX) analysis (Hitachi, Tokyo, Japan). Figure 8a shows the FESEM image and EDX mapping used to investigate the elemental distribution before the laundry cycle. The EDX results indicate the presence of carbon (C), oxygen (O), and silicon (Si). Notably, the treated fabric, PO-HS (2.0), exhibited a prominent presence of oxygen, which was consistent with the attachment of HS nanoparticles to the PET fabric. The presence of carbon was attributed to its composition within the PET polymer [48]. The FESEM images revealed agglomeration, which can be attributed to the strong affinity between the HS nanoparticles and the surface of the PET fiber. These agglomerates adhered to the fiber and contributed to an increase in surface roughness, thereby enhancing the surface hydrophobicity, as proposed by the Cassie–Baxter model [39]. Similar results were observed when the PO-HS(2.0) fabric was subjected to multiple washing cycles, as depicted in Figure 8b. It was noted that after the 20th cycle, no significant morphological changes were observed. However, it is noteworthy that a small fraction of HS nanoparticles (1.3 wt.%) was released during the washing step. The findings of this study indicate that the coating of the PET fabric with PO-HS (2.0) nanoparticles exhibited remarkable durability, as evidenced by the minimal detachment of HS particles even after multiple washing cycles. These results suggest that the fabric prepared with PO-HS (2.0) has the potential for various commercial applications owing to its excellent superhydrophobic properties and negligible loss of particles during use.

**Figure 9:**
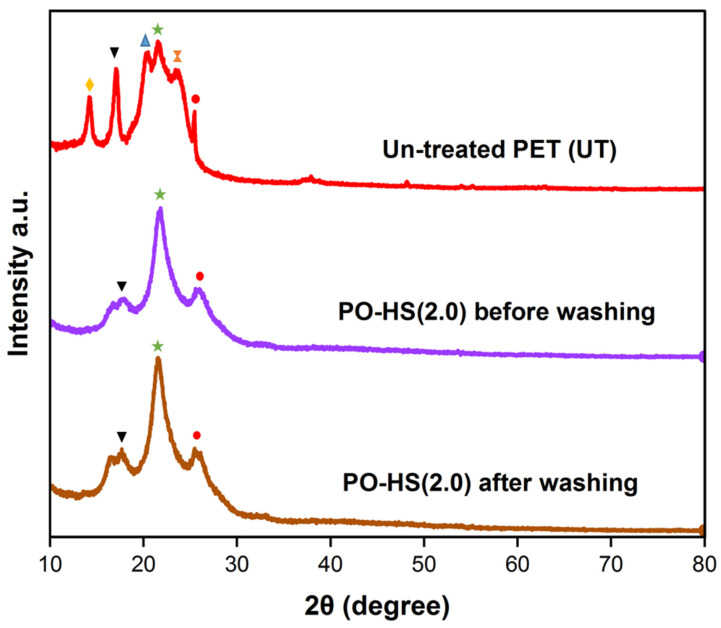
XRD patterns of untreated PET and PO-HS(2.0) fiber following treatment but before washing, and PO-HS(2.0) fiber after undergoing 20 cycles of washing.

Furthermore, we conducted an X-ray diffraction (XRD) analysis to examine the morphological changes in the untreated PET fabric and the treated sample, PO-HS(2.0), both before and after the 20th washing cycle, as shown in Figure 9. The XRD pattern of untreated PET fibers typically exhibited several distinct peaks, with the most prominent peak occurring at a 2θ value of 23.11°, followed by peaks at 14.05°, 17.10°, 21.09°, 20.31°, and 25.50°. These peaks are indicative of the polyester polymer present in PET, a confirmation supported by various studies [49,50,51,52,53]. After treating the original PET fiber with OTS and hollow silica nanoparticles to fabricate PO-HS(2.0), we observed a notable change in the XRD pattern. Specifically, the hollow SiO_2_ nanoparticles introduced a single broad peak centered at 2θ = 22.08°, suggesting the formation of a nanocrystalline SiO_2_ phase [54,55]. The sharp and elevated nature of this peak is attributed to the smaller particle size and hollow, incomplete inner structure of the spherical and smooth-surfaced SiO_2_ nanoparticles [53,56]. Notably, in the XRD profile of the hollow SiO_2_ NP-treated PET fiber, no additional peaks were observed, apart from those present in the raw PET fiber. The lack of unwanted peaks in the XRD profile of PO-HS(2.0) indicates that no new crystalline phases were formed during the chemical modification of the PET fiber. Similarly, after subjecting the treated fiber to 20 washing cycles, no discernible alterations were observed in the XRD pattern. This observation provides strong evidence for the effective coating of SiO_2_ and OTS on the surface of PET fibers, resulting in a highly rough surface with excellent superhydrophobicity. Finally, we conducted a comparative analysis of our findings with those of previously published studies, as outlined in Supplementary Table S3.

## 4. Conclusions and Future Direction

In summary, this study successfully developed a superhydrophobic water-repellent coating for PET fabrics using an environmentally friendly approach that employs an OTS solution through a dip-coating method. By incorporating HS nanoparticles into the coating compound, we enhanced the surface roughness and achieved exceptional water repellency, thus providing a sustainable alternative to fluorine-based compounds. We demonstrated the creation of hierarchical nanostructures and improved surface morphology by varying the weight percentages (0.5, 1.0, 1.5, and 2.0 wt.%) of HS nanoparticles. A thorough examination of the surface, chemical, thermal, and mechanical properties revealed that the inclusion of HS nanoparticles, especially at concentrations exceeding 1.0 wt.%, resulted in water contact angles exceeding 150°, indicating the attainment of superhydrophobic surfaces. Impressively, textiles coated with 2.0 wt.% HS exhibited contact angles up to 158.5°. This underscores the direct relationship between the HS content and textile hydrophobicity, allowing for customizable levels of water repellency. To assess the practicality of these superhydrophobic textiles, we subjected the optimal PO-HS(2.0) sample to 20 consecutive washing cycles, which revealed only a slight reduction in the water contact angle to 152.4° and the minimal detachment of the HS particles. This environmentally friendly, cost-effective, and relatively straightforward procedure has tremendous potential for imparting high hydrophobicity to PET fabric surfaces, making it applicable for various commercial uses. Future investigations should focus on ensuring the long-term stability of this technology when applied to diverse textile polymers, thereby enhancing its suitability for widespread commercial adoption. Furthermore, there is exciting potential for exploring new applications in the industrial sector such as oil repellency. This research represents a significant step towards sustainable and innovative advancements in textile technology, offering solutions to practical challenges and driving progress towards eco-friendly and efficient materials.

## Figures and Tables

**Table 1:** OTS and HS contents of the prepared samples.

Sample Name	Weight Ratio of Hollow SiO_2_ to OTS (wt.%)	
	OTS	HS
UT	-	-
PO	100	-
GO	100	-
PO-HS(0.5)	100	0.5
PO-HS(1.0)	100	1.0
PO-HS(1.5)	100	1.5
PO-HS(2.0)	100	2.0

**Table 2:** Water contact angles and residue values at 790 °C obtained from the TGA curves of the samples.

Specimens	Water Contact Angle (°)	Residue at 790 °C (%)
UT	-	9.4
GO	118.0 ± 3.8	-
PO	143.0 ± 8.2	9.2
HS	-	90.9
PO-HS(0.5)	149.5 ± 6.8	8.9
PO-HS(1.0)	153.0 ± 2.0	12.7
PO-HS(1.5)	156.5 ± 3.0	12.0
PO-HS(2.0)	158.5 ± 3.7	13.3

**Table 3:** Laundering cycle durability of the treated fabric and contact angle measurements obtained during 20 laundering cycles.

PO-HS (2.0)	Water Contact Angle (°)	Rate of Durability (° per Cycle)
1st cycle	158.3 ± 0.2	N/A
5th cycle	157.2 ± 0.6	0.275
8th cycle	156.5 ± 0.5	0.257
12th cycle	155.8 ± 0.3	0.227
16th cycle	153.9 ± 0.4	0.293
20th cycle	152.4 ± 0.8	0.312

## Data Availability

Data will be available on request.

## Supplementary Materials

The following supporting information can be downloaded at: https://www.mdpi.com/article/10.3390/polym15204065/s1, Figure S1: Illustrates the analysis of the particle size distribution of hollow silica nanoparticles (HS) using SEM and TEM images. Table S1: An overview of numerous varieties of hydrophobic/superhydrophobic coatings and their application methods. Table S2: Present an overview of FTIR results analysis results. Table S3: Presents a comparative analysis of hydrophobic and superhydrophobic coating by using OTS as a precursor and incorporating silica nanoparticles. Movie S1: Immersion of untreated PET fabric in rose Bengal dye solution. Movie S2: Immersion of treated PET fabric PO-HS(2.0) in rose Bengal dye solution. Movie S3: Slow-motion footage demonstrating the superhydrophobic behavior of treated PET fabric PO-HS(2.0) by adding droplets of rose Bengal dye solution.
