# Supplementary material for: Enhancing Textile Water Repellency with Octadecyltrichlorosilane (OTS) and Hollow Silica Nanoparticles
Source: Polymers (Basel). 2023 Oct 12;15(20):4065. doi: 10.3390/polym15204065 (PMC10610727; doi:10.3390/polym15204065)
Supplement: Supplementary file 1 [file polymers-15-04065-s001.zip › polymers-2631562-supplementary.pdf]

Supplementary Information

Enhancing Textile Water Repellency with Octadecyltrichlorosilane (OTS) and Hollow Silica Nanoparticles

Mahshab Sheraz [1], Byul Choi [1] and Juran Kim [1,2,*]

[1] Advanced Textile R&D Department, Korea Institute of Industrial Technology (KITECH), Ansan 15588, Republic of Korea; mahshab@kitech.re.kr (M.S.); bychoi@kitech.re.kr (B.C.)
[2] HYU-KITECH Joint Department, Hanyang University, 222, Wangsimni-ro, Seongdong-gu, Seoul 04763, Republic of Korea
* Correspondence: jkim0106@kitech.re.kr; Tel.: +82-10-5241-1923

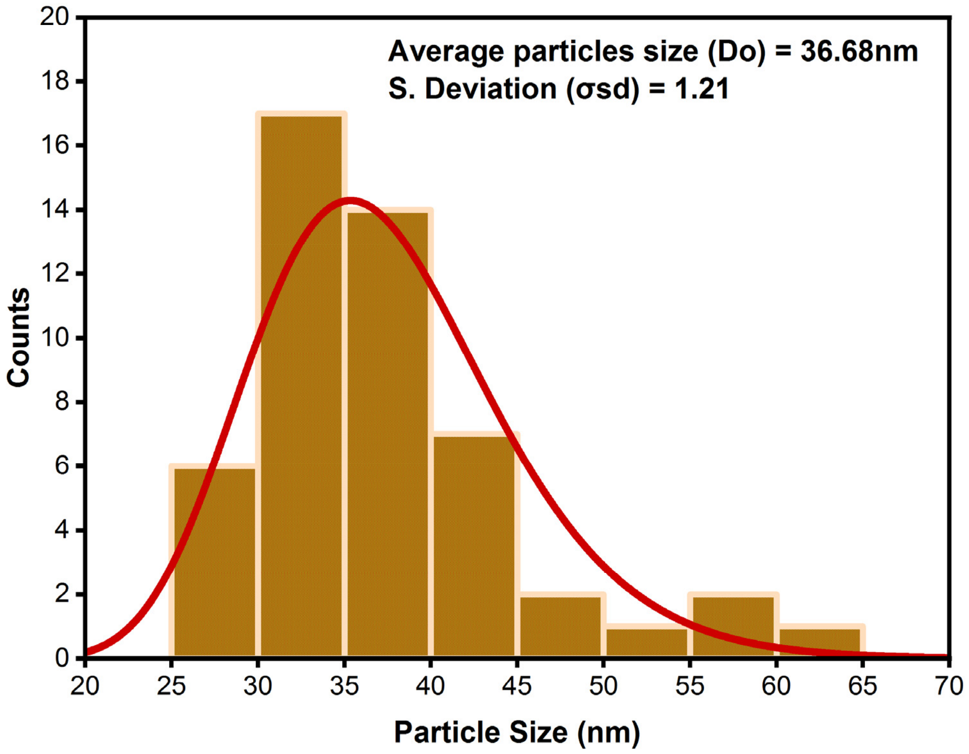

Figure S1. illustrates the analysis of the particle size distribution of hollow silica nanoparticles (HS) using SEM and TEM images.

Table S1. An overview of numerous varieties of hydrophobic/superhydrophobic coatings and their application methods.

Coatings	Methods	Description	Advantages	Disadvantages	References
Wax and resin mixture coatings	Spray method, Inlaying method, Finishing method	Surface treatments that provide a certain level of hydrophobicity while maintaining the permeability of fabric interstices.	Breathable and comfortable to wear	These treatments may exhibit reduced longevity and may necessitate periodic reapplication.	[1–3]
Aluminum salts coating	Treatment in aqueous solution, Physical vapor deposition (PVD), Spray method, Electroless plating method	Surface treatments that confer water-repellent properties.	Enduring and efficacious	Possible to effect fabric color or texture	[4–6]
Silicones coating	Dipping method, Spin coating, Solid silicone and solvent method, Silicone emulsions, vapor deposition.	Coating textile materials with a water-repellent layer via various methods	Effective and robust	Possible impact on fabric's permeability	[7,8]
Fluorochemicals coating	Dip coating method, Spray method, In-situ method, Sol-gel method	Utilized for achieving both hydrophobic (water-repelling) and oleophobic (oil-repelling) applications	Effective water and oil repellency	Could potentially raise environmental apprehensions or ecological considerations	[9,10]
Hydrophilic finishing agents(organosilane coatings)	Dip coating method, Spray method, Sol-gel process, Stoichiometric salinization	Used for water-repellent coatings. organosilane coating is commonly employed to produce a superhydrophobic surface on textiles, typically through methods such as spraying or dipping.	Excellent water repellency	This could potentially impact the breathability of the fabric.	[11–13]

Table S2. presents an overview of FTIR analysis results.

Functional group	Bond Type	Wavenumber range (cm^{-1})
Methylene group (symmetric and asymmetric stretching mode)	-CH2	2851 and 2919
Methylene group (symmetric and asymmetric stretching mode)	-CH3	2959
Carbonyl group (vibrational mode)	C=O	1715
Carbonyl group (stretching mode)	C-C-O, O-C-C	1243 and 1096
Vibration of aromatic rings	C-H	722
Silanol group asymmetric stretching vibration band	Si-O-Si	804

Table S3. presents a comparative analysis of hydrophobic and superhydrophobic coating by using OTS as a precursor and incorporating with silica nanoparticles.

Method	WCA	Major findings	Reference
Mixing a small quantity of water with a sufficient amount of bulk OTS according to stoichiometric proportions, then thinning the mixture and applying it to substrates through either immersion or spraying.	>170°	In summary, this superhydrophobic coating technique, achieved through precise organosilane aggregation control, offers a versatile and cost-effective approach for large-scale surface modifications in practical applications. It overcomes the limitations of existing waterproof coating methods and can be applied to diverse solid substrates. The created micro-to-nanoscale structures can also serve as templates for additional fabrications under ambient conditions.	[11]
SiO_2 nanoparticles are initially subjected to a surface modification process, wherein they are immersed in a solution containing OTS and toluene. Subsequently, these OTS-coated nanoparticles are incorporated into an alcohol-based solution, yielding a suspension that can be employed for spray deposition onto diverse substrates.	153°	In summary, superhydrophobic paper can be easily achieved by spraying an ethanol-based SiO_2 nanoparticle suspension onto paper at room temperature and atmospheric pressure. Hydrophobicity is controlled by nanoparticle aggregation (dependent on the alcohol used) and particle size. Importantly, text visibility remains unaffected. This method's key advantage lies in its simplicity, eliminating the need for expensive equipment, extreme conditions, or specialized nanomaterials to create superhydrophobic and transparent paper coatings.	[14]
SiO_2 nanoparticles undergo a process in which they are first treated with chlorotrimethylsilane (CTMS) and then with OTS. These modified SiO_2 particles are subsequently dispersed using ultrasound in a solution containing a polystyrene binder. The resulting mixture is then applied to a substrate through a drop-coating method.	156 ± 1°	This study developed superhydrophobic filters using a novel method that involved modifying SiO2 surfaces with two different alkylsilanes. Sequentially applying CTMS with short chains followed by OTS with longer chains increased the density of low-energy alkyl chains on the SiO2 surface, resulting in highly water-repellent surfaces with micro/nanoscale roughness. Filters made by coating cotton fabric with a CTMS + OTS-modified SiO_2/PS composite (2:1 weight ratio) achieved superhydrophobicity with a water contact angle of 156° ± 1°. These filters effectively repelled water, separated oil from water, and were reusable. This approach has the potential for creating similar superhydrophobic materials.	[15]

The process includes the synthesis of core-shell fluorescent silica nanoparticles, their application onto cotton fibers, a two-step silanization treatment of the fibers, and the curing of the silanized fibers to produce a superhydrophobic coating.	150°	In summary, this research showcases an uncomplicated and environmentally friendly approach to producing hydrophobic cotton fibers by utilizing silica nanoparticles and surface silanization. The resulting robust superhydrophobic coating remains resilient even after washing, suggesting a promising avenue for the development of water-repellent and corrosion-resistant cotton fabrics.	[16]
SiO_2 nanoparticles undergo a modification process involving the initial treatment with OTS. Afterward, they are combined with epoxy resin and polydimethylsiloxane in a solution of ethyl acetate. This mixture is subsequently sprayed onto the substrate.	159.5°	This study developed multi-functional wood materials with magnetic, superhydrophobic, and anti-ultraviolet properties. By combining $CoFe_2O_4$ nanoparticles and octadecyltrichlorosilane (OTS), the wood achieved a saturation magnetization of 1.8 emu/g and a coercivity of 450 Oe. It also exhibited superhydrophobicity with a high contact angle of around 150° and strong resistance to ultraviolet radiation. Overall, this approach enhances wood's functionality for diverse applications.	[17]
To create a SiO2 particle layer on a cotton substrate, the cotton is initially immersed in a TEOS solution. Subsequently, it is dipped into an OTS/ethanol solution for 2 hours.	151° ± 1.2°	A novel method using cotton fibers and the sol-gel technique was employed to create highly efficient superhydrophobic/oleophilic fibers for oil absorption and oil-water separation. This modification enhanced the cotton's ability to separate oil from water, allowing it to selectively absorb oil from oil-water mixtures and maintain its effectiveness over multiple uses. The modified cotton is a cost-effective, eco-friendly, and easily producible absorbent, making it suitable for large-scale oil spill cleanup on water surfaces.	[18]
To design an outstanding superhydrophobic PET fiber surface, we employ a dipping method incorporating OTS (octadecyltrichlorosilane) and hollow silica nanoparticles. Additionally, to check the performance of superhydrophobic surface the laundry washing tests were conducted to check its durability following multiple washing cycles.	152.4° ± 0.8°	This study successfully created a superhydrophobic coating for PET fabric using an eco-friendly approach involving OTS through dip-coating. Adding HS nanoparticles improved surface roughness and water repellency, offering a green alternative to fluorine-based coatings. Different HS nanoparticle concentrations (0.5%, 1.0%, 1.5%, and 2.0%) were used to enhance surface properties. Concentrations above 1.0% resulted in superhydrophobic surfaces with water contact angles above 150°, reaching 158.5% with 4.0% HS. Higher HS content led to increased hydrophobicity, and even after 20 washes, the optimal [PO-HS (2.0)] sample maintained a water contact angle of 152.4°. This eco-friendly and cost-effective method shows promise for commercial applications in creating highly water-repellent PET fabrics.	This work

References

1. Saji, V.S. Wax-Based Artificial Superhydrophobic Surfaces and Coatings. *Colloids and Surfaces A: Physicochemical and Engineering Aspects* **2020**, *602*, 125132, doi:10.1016/j.colsurfa.2020.125132.
2. Janesch, J.; Arminger, B.; Gindl-Altmutter, W.; Hansmann, C. Superhydrophobic Coatings on Wood Made of Plant Oil and Natural Wax. *Progress in Organic Coatings* **2020**, *148*, 105891, doi:10.1016/j.porgcoat.2020.105891.
3. Shen, T.; Fan, S.; Li, Y.; Xu, G.; Fan, W. Preparation of Edible Non-Wettable Coating with Soybean Wax for Repelling Liquid Foods with Little Residue. *Materials* **2020**, *13*, 3308, doi:10.3390/ma13153308.
4. Varshney, P.; Mohapatra, S.S.; Kumar, A. Superhydrophobic Coatings for Aluminium Surfaces Synthesized by Chemical Etching Process. *International Journal of Smart and Nano Materials* **2016**, *7*, 248–264, doi:10.1080/19475411.2016.1272502.
5. da Silva, R.G.C.; Malta, M.I.C.; de Carvalho, L.A.P.; da Silva, J.J.; da Silva Filho, W.L.C.; Oliveira, S.H.; de Araújo, E.G.; Urtiga Filho, S.L.; Vieira, M.R.S. Low-Cost Superhydrophobic Coating on Aluminum Alloy with Self-Cleaning and Repellency to Water-Based Mixed Liquids for Anti-Corrosive Applications. *Surface and Coatings Technology* **2023**, *457*, 129293, doi:10.1016/j.surfcoat.2023.129293.
6. Saleema, N.; Sarkar, D.K.; Paynter, R.W.; Chen, X.-G. Superhydrophobic Aluminum Alloy Surfaces by a Novel One-Step Process. *ACS Applied Materials & Interfaces* **2010**, *2*, 2500–2502, doi:10.1021/am100563u.
7. Li, Z.; Wang, X.; Bai, H.; Cao, M. Advances in Bioinspired Superhydrophobic Surfaces Made from Silicones: Fabrication and Application. *Polymers* **2023**, *15*, 543, doi:10.3390/polym15030543.
8. Ding, Z.; Lin, W.; Yang, W.; Chen, H.; Zhang, X. A Silicone Resin Coating with Water-Repellency and Anti-Fouling Properties for Wood Protection. *Polymers* **2022**, *14*, 3062, doi:10.3390/polym14153062.
9. Yin, X.; Sun, C.; Zhang, B.; Song, Y.; Wang, N.; Zhu, L.; Zhu, B. A Facile Approach to Fabricate Superhydrophobic Coatings on Porous Surfaces Using Cross-Linkable Fluorinated Emulsions. *Chemical Engineering Journal* **2017**, *330*, 202–212, doi:10.1016/j.cej.2017.06.145.
10. Khanjani, P.; King, A.W.T.; Partl, G.J.; Johansson, L.-S.; Kostiainen, M.A.; Ras, R.H.A. Superhydrophobic Paper from Nanostructured Fluorinated Cellulose Esters. *ACS Applied Materials & Interfaces* **2018**, *10*, 11280–11288, doi:10.1021/acsami.7b19310.
11. Zhang, L.; Zhou, A.G.; Sun, B.R.; Chen, K.S.; Yu, H.-Z. Functional and Versatile Superhydrophobic Coatings via Stoichiometric Silanization. *Nature Communications* **2021**, *12*, 982, doi:10.1038/s41467-021-21219-y.
12. Wang, X.; Lu, Y.; Zhang, Q.; Wang, K.; Carmalt, C.J.; Parkin, I.P.; Zhang, Z.; Zhang, X. Durable Fire Retardant, Superhydrophobic, Abrasive Resistant and Air/UV Stable Coatings. *Journal of Colloid and Interface Science* **2021**, *582*, 301–311, doi:10.1016/j.jcis.2020.07.084.
13. Liu, B.; Tian, T.; Yao, J.; Huang, C.; Tang, W.; Xiang, Z.; Xu, X.; Min, J. Superhydrophobic Organosilicon-Based Coating System by a Novel Ultravoilet-Curable Method. *Nanomaterials and Nanotechnology* **2017**, *7*, 184798041770279, doi:10.1177/1847980417702795.
14. Ogihara, H.; Xie, J.; Okagaki, J.; Saji, T. Simple Method for Preparing Superhydrophobic Paper: Spray-Deposited Hydrophobic Silica Nanoparticle Coatings Exhibit High Water-Repellency and Transparency. *Langmuir* **2012**, *28*, 4605–4608, doi:10.1021/la204492q.
15. Seeharaj, P.; Pasupong, P.; Detsri, E.; Damrongsak, P. Superhydrophobilization of SiO2 Surface with Two Alkylsilanes for an Application in Oil/Water Separation. *Journal of Materials Science* **2018**, *53*, 4828–4839, doi:10.1007/s10853-017-1925-5.
16. Maia, M.T.; Noronha, V.T.; Oliveira, N.C.; Alves, A.C.; Faria, A.F.; Martinez, D.T.S.; Ferreira, O.P.; Paula, A.J. Silica Nanoparticles and Surface Silanization for the Fabrication of Water-Repellent Cotton Fibers. *ACS Applied Nano Materials* **2022**, *5*, 4634–4647, doi:10.1021/acsanm.1c03346.
17. Gan, W.; Gao, L.; Sun, Q.; Jin, C.; Lu, Y.; Li, J. Multifunctional Wood Materials with Magnetic, Superhydrophobic and Anti-Ultraviolet Properties. *Applied Surface Science* **2015**, *332*, 565–572, doi:10.1016/j.apsusc.2015.01.206.
18. Lv, N.; Wang, X.; Peng, S.; Luo, L.; Zhou, R. Superhydrophobic/Superoleophilic Cotton-Oil Absorbent: Preparation and Its Application in Oil/Water Separation. *RSC Advances* **2018**, *8*, 30257–30264, doi:10.1039/C8RA05420G.
